# The Role of SBRT in Oligometastatic Prostate Cancer: Where We Are and Where We Are Heading

**DOI:** 10.3390/life16040550

**Published:** 2026-03-26

**Authors:** Macarena Teja, Miguel Angel Berenguer Frances, Fernando López Campos, Nicolas Feltes Benítez, Alexandra Stoica, Andrea Puertas, Giulia Marvaso, Vedang Murthy, Felipe Couñago

**Affiliations:** 1 Department of Radiation Oncology, GenesisCare-Vithas La Milagrosa University Hospital, 28010 Madrid, Spain; macarena.teja@genesiscare.es (M.T.);; 2 Department of Radiation Oncology, GenesisCare-San Francisco de Asís University Hospital, 28002 Madrid, Spain; 3 PhD Program in Medicine and Surgery, Escuela de Doctorado, Universidad Autónoma de Madrid, 28049 Madrid, Spain; 4 Department of Radiation Oncology, Vithas Consuelo Hospital, GenesisCare, 46007 Valencia, Spain; migberenguer@hotmail.com; 5 Department of Radiation Oncology, La Fe Hospital, 46206 Valencia, Spain; 6 Department of Radiation Oncology, Ramón y Cajal University Hospital, 28034 Madrid, Spain; 7 Department of Radiation Oncology, Terrassa University Hospital, 08221 Terrassa, Spain; nicofeltes_81@hotmail.com; 8 Department of Radiation Oncology, Torrejón University Hospital, 28850 Torrejón de Ardoz, Spain; alexandra.stoica@genesiscare.es; 9 Department of Radiation Oncology, GenesisCare Granada, 18008 Granada, Spain; 10 Division of Radiation Oncology, IEO European Institute of Oncology, Scientific Institute for Research, Hospitalization and Healthcare, 20141 Milan, Italy; giulia.marvaso@ieo.it; 11 Department of Oncology and Hematology-oncology, University of Milan, 20122 Milan, Italy; 12 Department of Radiation Oncology, Tata Memorial Centre, Homi Bhabha National Institute, Mumbai 400012, India; 13 Department of Medicine, School of Medicine, Health and Sport, European University of Madrid, 28670 Madrid, Spain

**Keywords:** oligometastases, prostate cancer, stereotactic body radiotherapy

## Abstract

Oligometastatic prostate cancer represents a distinct biological state between localized and widely metastatic disease, characterized by a limited number of lesions. Stereotactic body radiotherapy (SBRT) has emerged as a key metastasis-directed therapy (MDT), enabling precise ablation of metastatic lesions with minimal toxicity. Prospective clinical trials such as SABR-COMET, STOMP, ORIOLE, RADIOSA, and EXTEND have shown that SBRT delays disease progression, prolongs progression-free survival, and postpones the need for systemic therapy, while maintaining a favorable safety profile. Nevertheless, methodological limitations persist, including heterogeneity in defining oligometastatic disease, variability in dosing and fractionation, and the lack of predictive biomarkers. Ongoing phase III trials aim to validate the integration of SBRT with modern systemic therapies, including next-generation androgen receptor pathway inhibitors, to optimize clinical outcomes in hormone-sensitive and castration-resistant oligometastatic prostate cancer. This review summarizes current evidence, clinical applications, and future directions for SBRT in this patient population.

## 1. Introduction

Oligometastatic prostate cancer represents a clinical and biological intermediate state between localized and widely metastatic disease, characterized by a limited number of metastatic lesions, typically less than three to five. This scenario has gained increasing attention due to advances in imaging techniques, such as prostate-specific membrane antigen positron emission tomography (PSMA-PET), which enable the early and precise detection of oligometastatic lesions.

Stereotactic body radiotherapy (SBRT) has emerged as the cornerstone of metastasis-directed therapy (MDT), providing effective local ablation with minimal margins and strict protection of adjacent organs of interest. Prospective trials have shown that SBRT, either alone or in combination with hormonal therapy (HT), can delay the systemic progression, improve progression-free survival (PFS), and postpone the initiation of androgen-deprivation therapy (ADT), while maintaining low toxicity and high reproducibility.

Despite these promising results, the current evidence is limited by methodological heterogeneity, including variations in inclusion criteria, definitions of oligometastatic disease, dose and fractionation schemes, and the lack of validated predictive biomarkers. Ongoing phase III trials aim to address these limitations and integrate SBRT with modern systemic therapies, including androgen receptor pathway inhibitors (ARPIs) and, in selected cases, chemotherapy, to establish evidence-based standards of care.

This narrative review was conducted with the aim of summarizing the current clinical evidence in oligometastatic prostate cancer, highlighting its efficacy, safety, and future directions in multidisciplinary management. Electronic databases including Pubmed/MEDLINE were used. Emphasis was placed on randomized clinical trials and meta-analyses referring to SBRT in oligometastatic prostate cancer. Additional relevant publications were identified through manual screening of the reference lists of the selected articles. Only English-language publications were included. ClinicalTrials.gov was used to identify ongoing relevant trials.

## 2. SBRT: Technical and Radiobiologic Principles

SBRT is characterized by the delivery of high doses per fraction (6–20 Gy) over a limited number of sessions (1–5), with submillimetric geometric precision that enables effective tumor ablation while ensuring the strict protection of surrounding organs of interest. This technique integrates rigid immobilization, advanced motion management, daily image guidance radiotherapy (IGRT), and highly conformal planning using intensity-modulated radiation therapy (IMRT) or volumetric modulated arc therapy (VMAT), allowing the administration of biologically ablative doses with extremely steep dose gradients. Timmerman described this technical philosophy as an approach grounded in minimal margins and reproducible accuracy, both of which are essential for the safe delivery of ablative radiotherapy [1]. These principles are particularly relevant in oligometastatic prostate cancer, where SBRT has become a key MDT.

### 2.1. Advantages over Conventional Radiotherapy

Compared with conventional fractionation, SBRT offers distinct radiobiological and clinical advantages. High fractional doses allow the achievement of biologically effective dose (BED) values exceeding 100–150 Gy, which are associated with local control rates higher than 85–90%. This is especially advantageous for tumors with a low α/β ratio, such as prostate cancer (estimated at approximately 1.5), providing a strong biological rationale for hypofractionated or ablative approaches [2]. The short overall treatment duration limits accelerated repopulation and improves patient convenience. In other solid tumors, SBRT has demonstrated excellent outcomes, with local control rates approaching 98% [3]. These benefits have driven growing interest and the development of clinical trials exploring SBRT in the oligometastatic prostate cancer setting.

### 2.2. Planning, Dose Selection, and Radiobiological Principles

SBRT planning requires meticulous motion assessment, reproducible patient positioning, and reduced planning target volume (PTV) margins (2–5 mm). Common regimens include 1 × 20–25 Gy, 3 × 10–18 Gy, or 5 × 7–10 Gy, selected to achieve an ablative BED while respecting strict organs of interest tolerance constraints.

High doses per fraction induce a dense pattern of ionizations and complex deoxyribonucleic acid (DNA) damage that is inherently difficult to repair. In ablative regimens, DNA repair mechanisms become saturated, leading to enhanced cell death [4]. In addition, SBRT induces endothelial and microvascular damage, secondary tumor reoxygenation, and immunomodulatory effects, all of which contribute to the high rates of local control observed with this modality.

### 2.3. Normal Tissue Tolerance and Timmerman Constraints

SBRT requires strict side effect control, as even small tissue volumes exceeding the tolerance thresholds may result in severe complications. The classical Timmerman constraints define essential dose–volume limits to ensure safe treatment delivery (Table 1). Compliance with these parameters, combined with sharp dose gradients and reduced margins, allows SBRT to be delivered safely and effectively.

## 3. Current Clinical Evidence of SBRT in Oligometastatic Disease

In recent years, several studies have evaluated the role of SBRT as MDT in patients with oligometastatic disease (Table 2).

**Table 2:** Published evidence from randomized trials on SBRT in hormone-sensitive prostate cancer.

Study	N	Metastases	Imaging	Systemic Therapy	Primary Endpoint	Safety (SBRT Arms)
SABR-COMET [5,6]	99 (prostate 18%)	≤5 (mixed)	CT ± PET/CT	ADT permitted	OS: 27.2% vs. 13.6% at 8 years (*p* = 0.008)	Grade ≥ 3 ≈ 10% in SBRT arm
STOMP [7,8]	62	≤3 (bone/nodal)	Choline-PET/CT	Not permitted	ADT-free survival: 34% vs. 8% at 5 years (*p* = 0.06)	No Grade ≥ 3
ORIOLE [9]	54	≤3 (bone/nodal)	CT + bone scan (PSMA-PET subanalysis)	Not permitted	6-month PFS: 61% vs. 19% (*p* = 0.005)	No Grade ≥ 3
RADIOSA [10]	105	≤3 (bone/nodal)	Choline/PSMA-PET/CT	ADT (6 months)	PFS: 32.2 vs. 15.1 months (*p* = 0·001)	No Grade ≥ 3
EXTEND [11]	87	≤5 (mixed)	CT + bone scan/Fluciclovine F18-PET/CT	ADT +/−ARPIs	PFS: Median not reached vs. 15.8 months (*p* < 0.001)	Grade ≥ 3 ≈ 7% in SBRT arm
LUNAR [12]	87	≤5 (mixed)	PSMA PET/CT	Not permitted	PFS: 17.6 vs. 7.4 months; (*p* < 0.0001)	Grade 3 = 6.7%
RAVENS [13]	64	≤5 (bone/nodal)	Conventional imaging/molecular imaging	Not permitted	Composite-PFS: 11.8 vs. 10.5 months (*p* = 0.24).	Grade 3 = 17% in Ra223 + SABR

ADT = androgen deprivation therapy CT = computed tomography. OS = overall survival. PET = positron emission tomography. PFS = progression-free survival. PSMA = prostate-specific membrane antigen. SBRT = stereotactic body radiotherapy.

### 3.1. SABR-Comet: Proof of Concept for Ablative Treatment

The SABR-COMET trialconducted by Palma et al. [5], represents the cornerstone that initiated the modern era of MDT.

This randomized phase II study compared standard palliative management versus the same treatment plus SBRT in patients with ≤5 metastatic lesions from solid tumors.

Although only 16/99 patients had prostate cancer (2/33 [6%] in the control arm and 14/66 [21%] in the SBRT arm), this subgroup provided the first prospective signal that ablative control of limited metastatic disease could translate into meaningful survival benefits.

Across the overall population, SBRT achieved a local control rate exceeding 90%, a median overall survival (OS) of 41 months versus 28 months in the control arm (HR 0.57; *p* = 0.006), and a significant improvement in PFS. Grade ≥ 3 adverse events occurred in approximately 10% of patients.

Although exploratory in the prostate cancer subgroup, this trial established the biological and methodological rationale that eradication of metastatic lesions may delay systemic progression and improve long-term outcomes, paving the way for prostate-specific randomized studies, and its durability was reinforced by extended follow-up, showing improved 8-year OS (27.2% vs. 13.6%; HR 0.50; *p* = 0.008) and 8-year PFS (21.3% vs. 0.0%; HR 0.45; *p* < 0.001), with no new grade 3–5 toxicity signals [6].

### 3.2. STOMP and ORIOLE: Consolidating the MDT Paradigm in Prostate Cancer

The STOMP [7] and ORIOLE [9] trials provided the first prostate-specific prospective validation of MDT in oligometastatic hormone-sensitive prostate cancer (omHSPC). Both studies shared a common objective: to determine whether treating all visible metastases could delay the initiation of ADT.

The STOMP trial [7], a multicenter phase II study led by Ost et al., enrolled 62 patients with ≤ 3 metastases (bone or nodal). All patients in the STOMP trial underwent staging with choline PET computed tomography (CT). Patients were randomized to active surveillance or MDT using SBRT or metastasectomy.

At five years [8], the ADT-free survival was 34% versus 8% (median 21 vs. 13 months; *p* = 0.11), confirming that MDT can postpone HT without compromising safety. Local control of treated lesions reached 100%, and no grade ≥ 3 toxicity was observed.

These results provided the first prospective evidence that MDT can defer systemic therapy, sparing patients the metabolic, cardiovascular, and sexual adverse effects associated with LHRHa.

The ORIOLE trial [9] reinforced these findings in 54 patients with 1–3 metastases identified by conventional imaging (CT plus bone scan) and subsequently reassessed using PSMA PET. Patients were randomized to SBRT or observation.

At six months, disease progression occurred in 19% versus 61% (*p* = 0.005), while local control was 97%. Importantly, PSMA PET imaging revealed that treating all PET-positive lesions markedly reduced the development of new distant metastases, confirming the biological relevance of complete lesion ablation.

A subsequent pooled analysis of STOMP and ORIOLE [14] demonstrated a consistent improvement in PFS (11.9 vs. 5.9 months; HR 0.44; *p* = 0.001) and provided pioneering molecular insights.

Patients harboring DNA damage–repair (DDR) gene mutations (ATM, BRCA1/2, RB1, TP53) experienced shorter PFS but still derived benefit from MDT, suggesting that genomic profiling may refine patient selection and guide combinations with systemic or radiopharmaceutical therapies.

### 3.3. RADIOSA: Combining SBRT with Short-Course ADT

The RADIOSA trial [10] extended the MDT concept by adding short-term HT. In this open-label phase II study, 105 patients with ≤3 metastases detected predominantly by choline or PSMA PET were randomized to SBRT alone or SBRT plus 6 months of ADT, with stratification by lesion site (bone 40% vs. lymph node 60%).

Local control was 96% overall (98/102 patients), with six in-field recurrences (three in the SBRT group and three in the SBRT plus ADT group) and 50 out-of-field recurrences.

With a median follow-up of 31 months (IQR 16–36) in both groups, the combined approach doubled the clinical PFS (32.2 vs. 15.1 months; HR 0.43; *p* = 0.001) and significantly reduced out-of-field progression (37% vs. 61%). 

Treatment was well tolerated, with one genitourinary grade 3 adverse event as left ureter stenosis in the SBRT with ADT group. 

RADIOSA therefore provides the first prospective evidence that short-course ADT enhances the systemic efficacy of SBRT, establishing a foundation for combined-modality strategies in recurrent omHSPC.

### 3.4. EXTEND: Integrating SBRT into Modern Systemic Therapy

The EXTEND trial [11] (Extending Systemic Therapy by Targeting Oligometastatic Disease) represents the next step in therapeutic integration.

A total of 87 patients with oligometastatic prostate cancer (≤5 lesions) who had received HT for ≥2 months were randomized 1:1 to definitive radiation to all metastatic sites plus intermittent HT (*n* = 43) or to intermittent HT alone (*n* = 44).

The addition of SBRT significantly improved the PFS (median not reached vs. 15.8 months; HR 0.25; *p* < 0.001) and also improved eugonadal PFS, defined as the time to progression after testosterone recovery (median not reached vs. 6.1 months; HR 0.32; *p* = 0.03), with a median follow-up of 22.0 months (range, 11.6–39.2).

ARPIs were not used in 56% of patients in the combined therapy arm and 61% in the HT only arm. Most patients had received prior definitive local therapy to the prostate (72% overall).

There were no grade 4 or 5 adverse events. Six grade 3 events were reported: three in the combined therapy arm (in three patients) and three in the HT only arm (in two patients).

In this randomized clinical trial, the addition of metastasis-directed radiotherapy to intermittent HT significantly improved the PFS and eugonadal PFS compared with intermittent HT alone in men with oligometastatic prostate cancer.

An updated analysis reported in late 2025 [15] further showed that MDT combined with continuous ADT improved the radiologic PFS and castration-resistance-free survival compared with continuous ADT alone.

### 3.5. LUNAR: The Addition of Neoadjuvant ^177^Lu-PNT2002 to SBRT

The LUNAR trial [12] evaluated the role of administering two cycles of the PSMA-targeted radioligand Lutetium-177 (^177^Lu-PNT2002) before SBRT in oligorecurrent-HSPC. Here, 87 patients with 1–5 lesions identified by PSMA-PET and no ADT within 6 months before enrollment were included. The primary endpoint was PFS. At a median follow-up of 22 months, adding lutetium to SBRT significantly improved the PFS: 17.6 months (95% CI, 15–not reached) versus 7.4 months (95% CI, 6.0–13.5) with SBRT alone (HR 0.37; 95% CI, 0.22–0.61; *p* < 0.0001).

Local control was excellent, with in-field progression observed in 2% of the SBRT-alone arm versus 0% in the combined therapy group. Safety was generally favorable, with grade 3 adverse events limited to lymphopenia (4.8% in the SBRT group vs. 6.7% in the lutetium + SBRT group), and no grade 4–5 events were observed.

### 3.6. RAVENS: A Negative Study of Ra223 Combined with SBRT

The RAVENS phase II trial [13] proposed that the addition of the alpha-emitter radium-223 dichloride (Ra223) to MDT potentially eliminated subclinical bone disease in omHSPC and, therefore, prolonged the time to progression. With this aim, 64 metachronous omHSPC patients with 1 to 3 bone metastases on conventional imaging or 1 to 5 on molecular imaging were randomized to receive SBRT to the metastases +/− Ra223 administered within 2 weeks after SBRT. The previous ADT had to have finished 6 months before enrollment. The primary endpoint was the composite PFS. No improvement was found with the intensification strategy, with a median PFS of 11.8 months in the SBRT group versus 10.5 months in the combined therapy group (adjusted hazard ratio [aHR] 1.42; 95% CI, 0.79–2.56; *p* = 0.24).

The study revealed several noteworthy results regarding biomarkers. Pathogenic high-risk (“HiRi”) mutations (ATM, BRCA1/2, RB1, or TP53) were associated with poorer PFS (HR 5.95; 95% CI, 1.83–19.3; *p* = 0.003), whereas higher levels of unique productive T-cell receptor rearrangements at 3 months were independently prognostic of improved PFS regardless of the treatment arm (aHR 0.45; 95% CI, 0.21–0.96; *p* = 0.04). Overall, the treatment was well tolerated: 11% of patients experienced grade 3 treatment-related adverse events (no grade 4–5), with 6% in the SBRT arm compared to 17% in the SABR + Ra223 arm.

### 3.7. Global Safety and Clinical Impact

Across prospective trials, SBRT demonstrated an excellent safety profile, with scarce treatment-related grade ≥3 adverse events (0.3%; 95% CI: 0–1%) and high local control rates (estimated 2-year LC 97% [95% CI: 94–98%]) [16]. These results position SBRT not only as a definitive local treatment but also as a combinable strategy with other therapies.

Collectively, these studies confirm that modern image-guided SBRT effectively delays systemic therapy, reduces metastatic progression, and maintains long-term tolerability, positioning it as a central component of multidisciplinary management in oligometastatic prostate cancer.

## 4. Role of SBRT According to the Subtype of Oligometastatic Prostate Cancer Patient

### 4.1. Oligometastatic Disease States: Oligorecurrence, Oligopersistence, and Oligoprogression

As described before, oligometastatic disease represents a distinct biological state of metastatic spread characterized by a limited number of lesions, typically less than three to five, and has been proposed as an intermediate condition between localized disease and widespread metastasis [17,18]. However, the oligometastatic state is not homogeneous, and clinical scenarios differ according to the timing of metastatic onset and the response to systemic therapy.

Oligorecurrence refers to the reappearance of metastatic disease in an oligometastatic pattern following a treatment-free interval after a prior response to therapy [7].

Oligopersistence describes a condition in which metastatic lesions remain stable or partially responsive during ongoing systemic treatment [19].

Oligoprogression is defined by the progression of a limited number of lesions or the emergence of new metastases during active systemic therapy, a scenario in which local ablative treatments may allow continuation and prolongation of the current systemic regimen [7].

Accurate identification of these disease states is crucial for selecting patients who may benefit from MDT, particularly SBRT, as emphasized in international consensus statements [20]. Their integration with the HS or HR state in prostate cancer is also important.

### 4.2. Hormone-Sensitive Prostate Cancer: Integration of the Wolverine Meta-Analysis

The recently published WOLVERINE [21] individual patient data meta-analysis pooled 472 patients from randomized MDT trials, including STOMP [7] and ORIOLE [8].

With a median followup of 41 months, the meta-analysis demonstrated a robust and consistent improvement in PFS (HR ~0.44), a reduction in distant metastases and radiographic progression, and a borderline trend toward improved OS (*p* = 0.051 in the random-effects model). Importantly, the benefit of MDT was observed across imaging modalities, metastatic burden (≤3 and ≤5 lesions), and in both de novo and metachronous disease presentations.

WOLVERINE strengthens the current evidence base, confirming that SBRT-based MDT provides clinically meaningful and durable disease control in omHSPC and should be considered a standard component of modern multimodal management.

### 4.3. Hormone-Sensitive Prostate Cancer: Combination of SBRT with Systemic Therapy

The RADIANT trial [22] was a prospective single-arm phase II study enrolling patients with HSPC experiencing oligoprogression while receiving HT, including conventional ADT or next-generation ARPIs. All metastatic lesions were treated with SBRT. The primary endpoint was the delay in systemic treatment modification, with 55% of patients remaining on the same systemic therapy at one year.

The PERSIAN trial [23] was a randomized phase II study evaluating the addition of metastasis-directed SBRT to ADT plus apalutamide in patients with oligometastatic HSPC (≤5 non-visceral metastases). The primary endpoint was the complete biochemical response rate at six months, with secondary endpoints including radiographic PFS and clinical outcomes.

The interim results presented at ASCO GU 2025 showed no significant difference in early biochemical response in the overall population; however, a benefit favoring the SBRT arm was observed in patients with low metastatic burden (≤3 lesions). These findings suggest that metastasis-directed therapy may provide additional benefit in selected patients receiving modern systemic treatment.

Overall, the current evidence largely derived from phase II trials suggests that combined treatment strategies incorporating SBRT with HT may provide improved oncological control in selected patients with oligometastatic prostate cancer.

### 4.4. Hormone-Resistant Prostate Cancer

In the setting of castration-resistant prostate cancer, three trials should be mentioned (Table 3): GROUQ-PCS 9 [24], ARTO [25] and TRAP [26]. In the first trial, enzalutamide played a central role. Patients who had progressed after ADT and had not previously received an ARPI were included. The two arms were ADT + ARPI (enzalutamide) versus ADT + ARPI (enzalutamide) + SBRT to metastases. Outcomes were superior in the experimental arm where SBRT was added, with a radiographic PFS of 4.6 years versus 2.3 years (*p* = 0.014). Improvements were also observed in the time to next systemic therapy and biochemical PFS.

**Table 3:** Published evidence from randomized trials on SBRT in castration-resistant prostate cancer.

Study	Year/Source	N	Metastatic Sites	Imaging	Local Control	Progression-Free Outcomes	Toxicity/Safety
GROUQ-PCS9 [24]	2025	102	≤5 mixed (bone ± node 85%)	Conventional imaging	100%	PFS 4.6 vs. 2.3 years	No G3 differences (%); no G4–5
		ADT + Enzalutamide vs. ADT + Enzalutamide +SBRT					
ARTO [25]	2023	157 (AAP + ADT vs. AAP + ADT + SBRT)	≤3 bone/nodal	Conventional + PSMA-PET	100%	SBRT (HR. 035)	No G ≥ 3; excellent tolerance

ADT = androgen deprivation therapy. PET = positron emission tomography. PFS = progression-free survival. SBRT = stereotactic body radiotherapy. PSMA = prostate-specific membrane antigen.

In the second trial, another phase II randomized study, the response to adding SBRT to metastases was evaluated in combination with abiraterone–prednisone (AAP) versus AAP alone in patients with oligometastatic castration-resistant prostate cancer.

The results showed that SBRT combined with AAP reduced the risk of progression by 65% compared with AAP alone (HR 0.35; *p* < 0.001). Additionally, the proportion of patients achieving PSA reduction ≥50% was higher with AAP + SBRT (92% vs. 68.3%).

The TRAP [26] trial was a prospective nonrandomized phase II study that demonstrated the feasibility and clinical activity of metastasis-directed SBRT in patients with oligoprogressive castration-resistant prostate cancer, achieving meaningful disease control and delaying treatment escalation with an acceptable toxicity profile. These findings provided proof of concept that ablative treatment of resistant metastatic lesions may enhance disease control while preserving ongoing systemic therapy.

Building on this rationale, the STAR-TRAP study is a multicenter randomized phase II trial assessing the addition of metastasis-directed SBRT to standard systemic therapy in patients with metastatic prostate cancer with limited disease burden. Patients are randomized to receive systemic therapy alone or in combination with SBRT delivered to all detectable metastatic lesions identified by modern imaging techniques.

The primary endpoint of STAR-TRAP is radiographic progression-free survival, with secondary endpoints including time to next systemic treatment, biochemical progression, toxicity, and quality of life. STAR-TRAP aims to provide prospective randomized evidence to validate the integration of metastasis-directed therapy into the management of advanced prostate cancer.

## 5. Critical Evaluation of Current Evidence

Despite the favorable findings reported across multiple studies, important methodological limitations and potential sources of bias remain within the current body of evidence.

First, most trials have been conducted in Western countries, which may limit their external validity and generalizability to other populations. Moreover, the available evidence largely derives from phase II clinical trials with small patient cohorts [7,9,10,11,24,25,27,28], some of which include patients with heterogeneous solid tumor histologies [5,29]. The study designs are highly heterogeneous, encompassing differences in inclusion criteria, diagnostic imaging modalities, treatment strategies, and primary endpoints. In addition, there is a lack of validated predictive biomarkers to assist clinicians in patient selection.

The definition of oligometastatic disease varies across studies, with the number of lesions ranging from three to five. These differences can influence studies outcomes, as trials with stricter criteria tend to include patients with lower disease burden and potentially better prognosis, whereas broader definitions allow inclusion of patients with more extensive oligometastatic disease. It is therefore essential to consider these definitional variations, as they can affect both the applicability of the results and cross-trial comparisons. The consensus from the European Society for Radiotherapy and Oncology (ESTRO) and the European Organisation for Research and Treatment of Cancer (EORTC) classified oligometastatic disease into nine subgroups with potential prognostic differences [30]. The classification first distinguishes between a genuine scenario, in which patients have no previous history of polymetastases, and the induced oligometastatic subgroup. Within the genuine state, patients are further categorized into de novo oligometastatic disease, when oligometastases are diagnosed for the first time, and repeat oligometastatic disease. The de novo scenario is further divided into synchronous and metachronous states, depending on whether the metastases appeared within or more than 6 months after the diagnosis of the primary tumor. Additional subgroups are established based on the presence of systemic therapy at the time of progression and on whether the lesions represent persistence or true progression of disease. Given the potential prognostic differences among these subgroups, their inclusion within the same study may introduce bias making it important to distinguish between them in future studies.

Although most trials distinguish between hormone-sensitive and castration-resistant prostate cancer, some include both populations. Given the markedly different prognoses of these disease states, failure to adequately stratify patients may confound outcomes. Furthermore, the use of PET-CT imaging was not mandatory in most trials; only the STOMP trial [7] required choline PET-CT, and none mandated PSMA PET-CT prior to enrolment. This may have introduced staging bias, as demonstrated in ORIOLE [9], where patients staged using conventional imaging had worse outcomes when additional untreated metastases were subsequently identified on PSMA PET-CT. Different retrospective [31] and prospective [32,33] trials have shown superior lesion detection rates of PSMA PET when compared with conventional imaging. Further, for PSA ≤ 1 ng/mL, a meta-analysis [34] and a systematic review [35] showed a higher lesion detection rate with PSMA PET compared to choline PET/CT. These results were confirmed by the phase III study of Panagiotidis et al. [36]. For these reasons, given the staging methods used in the available clinical trials, the results should be interpreted with caution due to the potential bias in metastasis detection. The improved sensitivity of molecular imaging and, especially, PSMA PET, may influence the outcomes differences between previous studies and recent ones, and this should be taken into account when interpreting the results of the phase III clinical trials that are still ongoing.

Regarding treatment strategies, the SBRT dose and fractionation schedules vary substantially across studies, complicating the standardization of radiotherapy regimens. Globally, 30 Gy in three fractions was the most common treatment scheme for bone or nodal SBRT. However, doses and fractionations ranged from single-fraction treatments of 16–24 Gy to 27–36 Gy in three fractions or 30–50 Gy in five fractions. For bone metastases, regimens around 27–36 Gy in three fractions or 30–40 Gy in five fractions were commonly used, whereas doses for visceral lesions typically ranged between 35 and 50 Gy in five fractions. Despite the heterogeneity, doses and fractionations are aligned with the recommendations of the main clinical practice guidelines. The ESTRO guidelines for spinal metastases recommend delivering a BED_10_ of at least 50 Gy [37]. More specifically, for bone metastases, international guidelines [38,39,40] propose 20–24 Gy in a single fraction, 27–30 Gy in three fractions and 30–35 Gy in five fractions. For lung and liver metastases, higher doses are advisable (BED_10_ > 100 Gy) [41,42].

Additionally, most studies involving hormone-sensitive oligometastatic patients did not incorporate contemporary standard-of-care systemic therapies, relying instead on ADT alone or observation [7,9,10,28]. This is a critical limitation, as the current evidence positions ARPIs [43,44,45,46,47,48,49] and in selected cases chemotherapy [50,51,52] as first-line treatment in this population. In the castration-resistant setting, GROUQ-PCS 9 [24] and ARTO [25] incorporated ARPIs into treatment strategies; however, the latter trial was terminated early due to slow accrual. Clinical practice guidelines recommend ADT with ARPIs as first line treatment for oligometastatic prostate cancer patients and the addition of MDT in selected cases but not without systemic therapies [53,54].

Importantly, outcome definitions are inconsistent across trials. Progression-free survival is variably defined and includes different event criteria. Primary endpoints also differ substantially: ADT-free survival in STOMP, the biochemical response rate in ART, and local control in SBRT-SG 05, while other trials use diverse PFS-based endpoints.

Collectively, the limitations outlined above underscore the need for further clarification to enable standardization of treatment protocols and the development of evidence-based clinical practice guidelines.

## 6. Future Directions

To address several of the limitations identified in earlier studies, multiple phase III and some relevant phase II trials evaluating the role of SBRT directed at metastatic sites are currently underway, incorporating important methodological innovations. Table 4 provides an overview of the most relevant ongoing studies in patients with oligometastatic prostate cancer.

In the setting of oligometastatic hormone-sensitive prostate cancer, some of the most relevant ongoing trials include the phase III START-MET (NCT05209243) [55], METRO (NCT04983095) [56], Oligo-PRESTO (NCT04115007) [57], STAMPEDE2 (NCT06320067) [58], PLATON (NCT03784755) [59] and the phase II METANOVA (NCT06150417) [60] and TERPS (NCT05223803) [61]. Notably, the first two studies mandate the use of molecular imaging (choline PET-CT or PSMA PET-CT) for staging prior to treatment [55,56]. Nevertheless, a degree of heterogeneity persists among the ongoing phase III trials with respect to imaging requirements.

Importantly, in all studies, patients in both treatment arms receive optimal systemic therapy, including ARPIs, rather than observation or ADT alone. Local treatment of the primary tumor, when not previously administered, is also a key component of all protocols, even in patients who do not meet traditional low-volume disease criteria. Consequently, if these trials demonstrate favorable outcomes, a multimodal approach combining SBRT to oligometastatic lesions, definitive local treatment of the primary tumor, and next-generation anti-androgens plus ADT may become the standard of care for patients with oligometastatic hormone-sensitive prostate cancer. Moreover, METANOVA and METRO trials further explore treatment de-escalation by limiting systemic therapy to 12 months in the first study and three years of ADT with two years of abiraterone–prednisone in the second one.

In the context of castration-resistant oligometastatic prostate cancer, both phase II and phase III trials are ongoing and incorporate additional methodological innovations. The PEACE8 trial (NCT06276465) [62] investigates whether the addition of SBRT to oligometastatic lesions improves outcomes compared with darolutamide plus ADT alone. The phase II PILLAR trial (NCT03503344) [63] compares SBRT combined with apalutamide versus apalutamide monotherapy. Notably, both studies require next-generation PET-CT imaging for metastatic disease detection and treatment planning. Treatment discontinuation strategies represent another innovative aspect of these trials, with darolutamide administered for up to five years in PEACE8 and apalutamide for 52 weeks in PILLAR.

Finally, several trials enrolling oligometastatic patients across different tumor histologies, including prostate cancer, warrant mention. Among the most relevant are STEREO-OS (NCT03143322) [64], SABR-COMET-10 (NCT03721341) [65], SABR-COMET-3 (NCT03862911) [66], and SABR-SYNC (NCT05717166) [67], all of which are currently awaiting results. Of particular interest, SABR-COMET-10 introduces a key novel feature by expanding the allowable number of metastatic lesions to up to ten, thereby challenging the traditional definition of oligometastatic disease.

**Table 4:** Ongoing clinical trials evaluating SBRT in oligometastatic prostate cancer ordered by hormone sensitivity status and estimated study completion date.

Study	Phase	Hormone Sensitivity Status	Primary Endpoint	Treatment Groups	Number of Metastases Allowed	Imaging Test	Metastasis Location	Study Completion Estimated
START-MET [55]	III	Hormone-Sensitive	rPFS	1. SBRT + RT to the primary (if not treated) + ARPI + ADT 2. RT to the primary (if not treated) + ARPI + ADT	5	CT + Choline PET-CT/PSMA PET-CT	Bone	2027
TERPS [61]	II	Hormone-Sensitive	2-year FFS	1. SBRT + RT to the primary + best systemic therapy (per oncologist decision) 2. RT to the primary + best systemic therapy (per oncologist decision)	3 (traditional imaging) 5 (functional imaging)	CT/MRI/Bone-scan or Fluciclovine/choline/PSMA PET-CT	Bone or soft tissue (at least one bone metastasis)	2027
METANOVA [60]	II	Hormone-Sensitive	FFS	1. SBRT + local therapy to the primary + ARPI + ADT 2. Local therapy to the primary + ARPI + ADT	5 (traditional imaging) 10 (PSMA PET-CT)	MRI, CT/99mTc bone scan or PSMA PET-CT	Bone, extra-pelvic lymph node	2028
PEACE 6 Oligo-PRESTO [57]	III	Hormone-Sensitive	Castration-resistant prostate cancer-free survival	1. SBRT+ RT to the primary (if not treated) + ARPI/docetaxel + ADT 2. 177Lu-PSMA-617 + RT to the primary (if not treated) + ARPI/docetaxel + ADT 3. RT to the primary (if not treated) + ARPI/docetaxel + ADT	5	Choline PET-CT/PSMA PET-CT/whole body MRI	Bone, lymph node, visceral	2031
STAMPEDE2 [58]	III	Hormone-Sensitive	OS	1. SBRT + RT to the primary (if not treated) + ARPI/docetaxel + ADT 3. 177Lu-PSMA-617 + RT to the primary (if not treated) + ARPI/docetaxel + ADT 2. RT to the primary (if not treated) + ARPI/docetaxel + ADT	5	CT/MRI and either bone or PET scan	Bone, lymph node	2032
METRO [56]	III	Hormone-Sensitive	FFS	1. SBRT + RT to the primary (if not treated) + ARPI + ADT 2. RT to the primary (if not treated) + ARPI + ADT	3	PSMA-PET-CT	Bone, extra pelvic lymph node	2033
PLATON [59]	III	Hormone-Sensitive	FFS	1. SBRT/Surgery + ablative treatment of the primary (if not treated) + Standard systemic therapy 2. Ablative treatment of the primary (if not treated) + Standard systemic therapy	5 (≤3 non-bone)	CT/MRI + Bone scan	Bone, lymph node, visceral	2033
PILLAR [63]	II	Hormone-Resistant	Undetectable PSA	1. SBRT + apalutamide 2. Apalutamide	5	PSMA-PET-CT	Bone, lymph node, visceral	2027
GETUG-AFU 43–PEACE8 [62]	III	Hormone-Resistant	rPFS	1. SBRT + darolutamide + ADT 2. Darolutamide + ADT	5	Choline, fluciclovine, or PSMA-PET-CT	Bone, lymph node (including pelvic), visceral	2032

ADT = androgen deprivation therapy. ARPI = androgen receptor pathway inhibitor. CT = computed tomography. FFS = failure-free survival. MRI = magnetic resonance imaging. OS = overall survival. PET = positron emission tomography. PSA = prostate-specific antigen. PSMA = prostate-specific membrane antigen. rPFS = radiological progression-free survival. RT = radiotherapy. SBRT = stereotactic body radiotherapy.

## 7. Conclusions

SBRT has proven to be an effective and safe strategy in the management of oligometastatic prostate cancer, both in hormone-sensitive and castration-resistant patients. Recent prospective trials and meta-analyses have demonstrated that SBRT, either alone or in combination with modern systemic therapies such as ARPIs, can delay disease progression, extend PFS, and postpone the need for HT, while maintaining a favorable toxicity profile.

However, the current evidence remains limited by methodological factors, including heterogeneity in the definition of oligometastatic disease, variability in dose and fractionation schemes, and the lack of validated predictive biomarkers. The results from ongoing phase III trials will be crucial to establish standardized treatment protocols, define patient selection criteria, and determine the optimal duration of systemic therapy in combination with SBRT.

In conclusion, SBRT represents a cornerstone in the multidisciplinary management of oligometastatic prostate cancer, offering effective local control, prolonged PFS, and the potential for integration with next-generation systemic therapies. Incorporating SBRT into multimodal protocols has the potential to transform the management of this patient population, optimizing clinical outcomes and quality of life.

## Figures and Tables

**Table 1:** Classic Timmerman dose constraints for SBRT [1].

Organ at Risk	1 Fraction (Gy)	3 Fractions (Gy)	5 Fractions (Gy)	Clinical Relevance
**Spinal cord**	10–14	17–22	23–28	Prevent radiation myelopathy
**Esophagus**	11	27.5	35	Risk of ulceration/perforation
**Trachea/Main bronchus**	12.4	30	40	Risk of stenosis/necrosis
**Bowel/Small intestine**	12.4	30	35	Perforation risk at hotspots
**Brachial plexus**	—	24	32–35	Prevent plexopathy
**Chest wall/Ribs**	—	V30 >30 cm^3^	V40 >30 cm^3^	Fracture and chronic pain
**Liver (functional reserve)**	≥700 cm^3^ < 9 Gy	≥700 cm^3^ < 15 Gy	≥700 cm^3^ < 21 Gy	Preserve hepatic function
**Kidney**	—	V16 < 33%	V12 < 55%	Renal safety

Gy = Gray.

## Data Availability

No new data were created or analyzed in this study. Data sharing is not applicable to this article.

## Abbreviations

The following abbreviations are used in this manuscript:

AAP	Abiraterone–prednisone
ADT	Androgen deprivation therapy
ARPIs	Androgen receptor pathway inhibitors
BED	Biologically effective dose
CT	Computed tomography
DDR	DNA damage–repair
DNA	Deoxyribonucleic acid
ESTRO	European Society for Radiotherapy and Oncology
EORTC	European Organisation for Research and Treatment of Cancer
HR	Hormone-resistant
HS	Hormone-sensitive
HT	Hormone therapy
IGRT	Image guidance radiotherapy
IMRT	Intensity-modulated radiation therapy
MDT	Metastasis-directed therapy
MRI	Magnetic resonance imaging
omHSPC	Oligometastatic hormone-sensitive prostate cancer
OS	Overall survival
PFS	Progression-free survival
PET	Positron emission tomography
PSA	Prostate-specific antigen
PSMA	Prostate-specific membrane antigen
SGRT	Surface-guided radiotherapy
SBRT	Stereotactic body radiotherapy
VMAT	Volumetric modulated arc therapy
